# Tissue Dimensionality Influences the Functional Response of Cytotoxic T Lymphocyte-Mediated Killing of Targets

**DOI:** 10.3389/fimmu.2016.00668

**Published:** 2017-01-11

**Authors:** Saikrishna Gadhamsetty, Athanasius F. M. Marée, Rob J. de Boer, Joost B. Beltman

**Affiliations:** ^1^Theoretical Biology, Utrecht University, Utrecht, Netherlands; ^2^Department of Computational and Systems Biology, John Innes Centre, Norwich, UK; ^3^Division of Toxicology, Leiden Academic Centre for Drug Research, Leiden University, Leiden, Netherlands

**Keywords:** CTL, tissue dimensionality, killing rate, cellular Potts model, functional response

## Abstract

Cytotoxic T lymphocyte (CTL)-mediated killing of virus infections and tumors occurs over a wide range of conditions. The spatial environments in which CTLs encounter target cells vary from narrow vessels, to two-dimensional epithelial tissues, to densely populated 3-dimensional (3D) T cell areas within lymphoid tissues. How such spatial environments alter the functional response of CTL-mediated killing, i.e., how the killing efficiency depends on cell densities, is unclear. In this study, we perform cellular Potts model simulations in different spatial configurations to investigate how the dimensionality of the space affects the functional response of CTL-mediated killing. Irrespective of the spatial configuration, the function with separate saturation constants for CTL and for target cell densities that we previously proposed can in all cases describe the response, demonstrating its generality. However, the tissue dimensionality determines at which cell densities the killing rate starts to saturate. We show that saturation in a fully 3D environment is stronger than in a “flat” 3D environment, which is largely due to accompanying differences in the CTL–target encounter rates.

## Introduction

Cytotoxic T lymphocytes (CTLs) continuously search for and kill virus-infected cells in tissues throughout our bodies. For example, CTLs specifically recognizing human immunodeficiency virus (HIV) epitopes colocalize with HIV-infected cells in the subcapsular sinuses of lymph nodes (LNs) (1), whereas CTL interactions with peptide-pulsed B cells predominantly occur in T cell areas in the cortex of LNs (2). Furthermore, tissue-resident memory T cells continuously patrol the skin epidermis to find targets (3, 4), and tumor-infiltrating CTLs navigate through spatially complex environments (5, 6). Therefore, it is important to understand how local tissue environments influence the CTL-mediated killing rates of target cells.

Analogous to its definition in ecology, the functional response of CTL-mediated killing is the rate at which a single CTL kills targets, as a function of the CTL and the target cell densities. The total killing rate at which target cells are killed is given by the product of CTL density and the functional response. Computer simulation studies have hitherto studied the functional responses of CTL-mediated killing in either 2D or 3D environments (7, 8). For example, we performed 2D cellular Potts model (CPM) (9, 10) simulations of CTL-mediated killing in a densely packed cellular environment mimicking T cell areas of an LN (8). Regardless of the CTL–target cell interactions, CTL-mediated killing was well described by a double saturation (DS) model with two saturation constants, which are defined as the CTL and target cell densities at which killing reaches half the maximal rate [see Methods and Ref. (8)]. Additionally, we analytically derived this DS model for cases where target cells are killed by a single CTL. For other cases, the double saturation model still provides a semi-mechanistic description, having three parameters with a sound biological meaning (8). However, the quantitative effects of tissue dimensionality on the functional response are still unknown and may be affected by factors such as search efficiency.

In the current study, we investigate how dimensionality of the tissue influences the functional response. To this end, we perform 3D CPM simulations of CTL killing in either a flat or a cubic spatial configuration. As for the published 2D simulations, the DS model appears valid for different types of CTL–target interactions in 3D (8). Moreover, we find that the tissue dimensionality affects the density at which the killing efficiency starts to saturate, predominantly due to differences in CTL–target encounter rates. Taken together, our results demonstrate that the double saturation model is a generic functional response and that spatial dimensionality plays a hitherto unrecognized role in determining the extent of saturation of CTL-mediated killing.

## Materials and Methods

### Model Description

We simulate a region of a spleen or a lymph node using the CPM formalism, in which each biological cell consists of multiple connected lattice sites (9, 10). We consider a 3D field composed of fibroblastic reticular cells forming a reticular network (RN; ≈24% of the field), 2,500 target cells (37%), 2,500 CTLs (37%), and extracellular matrix (≈3%). Changes in the cell configuration and movements of the cells occur due to minimization of the surface energy of the cells. Within each time step, all positions on the lattice are considered for extension into a random neighboring site, and the change in surface energy due to an extension is calculated by the difference in Hamiltonians *H* of two configurations. The Hamiltonian is given by

(1) $$\begin{array}{l} H=\sum_{ijk}\;\sum_{i^{\prime }j^{\prime }k^{\prime }}\;J_{\tau \left(\sigma_{\text{ijk}}\right),\tau \left(\sigma_{i\prime j\prime k\prime }\right)}\left(1-\delta_{\sigma_{\text{ijk}},\sigma_{i\prime j\prime k\prime }}\right)+\sum_{\sigma }\;\lambda {\left(v_{\sigma }-V_{\tau \left(\sigma \right)}\right)}^{2}, \end{array}$$

where $J_{\tau \left(\sigma_{\text{ijk}}\right),\tau \left(\sigma_{i\prime j\prime k\prime }\right)}$ is the surface energy associated with the neighboring lattice sites and *δ* is the Kronecker delta. The first term in the above equation represents the sum of all surface energies, and the second term is a volume constraint applied to maintain the size of the cells close to their target volume, *V*_τ__(__*σ*__)_; τ(*σ*) is the cell type of the cell with identification number *σ*; and λ is the inelasticity. The probability that a lattice site is copied into the neighboring site is one if Δ*H* < 0, and *e*^−Δ^*^H/T^* otherwise, where *T* represents the membrane fluctuation amplitude of cells. The details of the simulation protocol, including the migration and killing algorithms, are described in full detail elsewhere (8). The entire model is implemented in the C programming language.

### Default Model Parameters

The CPM phenomenologically describes the cell migration and interaction behavior. The CPM simulation parameters, including the surface energies and adhesion parameters, are chosen such that we approximate the migration properties of CTLs and target cells (B cells, in our case) observed *in vivo* (2, 11). Thus, these parameters have no direct biological meaning. We use the following parameters described for all simulations in this study, unless otherwise specified. We consider two 3D fields of similar volume 107 *μ* m × 107 *μ*m × 107 *μ*m and 350 *μ*m × 350 *μ*m × 10 *μ*m, where the length of each position on the lattice equals 1 *μ*m. Parameters are chosen such that one time step in the simulation (i.e., attempting to update all the lattice sites) corresponds to 1 s in real time. To maintain similar migration properties at different frequencies of CTLs and target cells, we vary the number of antigen-expressing target cells, $\bar{T}$, and their cognate CTLs, $\bar{E}$, while keeping the total number of CTLs and target cells in the field at a constant value of 5,000 cells. Following the initialization of the RN, both target cells and CTLs are initialized at empty random positions as a cube of 27 *μ*m^3^, which subsequently grow to their target volume of 180 *μ*m^3^, corresponding to a diameter of about 7 *μ*m (12, 13). We use a kill time (i.e., the time required for a CTL to induce a target cell death), *t_D_*, of 15 min (2). The default surface energy values and the adhesion strengths used in the simulations are shown in Table 1. Other default parameters used in both configurations: directional propensity, *μ* = 1,150 for CTLs, *μ* = 850 for target cells; inelasticity of cells, λ = 350, and membrane fluctuation amplitude, *T* = 100.

**Table 1:** **Default surface energies, *J*, and surface tensions, γ, used in the simulations of both spatial configurations**.

	ECM	RN	CTL	Tgt
ECM	*J_ECM, ECM_* = 0	γ*_ECM, RN_* = 0	γ*_ECM, CTL_* = 0	γ*_ECM, Tgt_* = 0
RN	*J_RN, ECM_* = 0	*J_RN, RN_* = 0	γ*_RN, CTL_* = 175	γ*_RN, Tgt_* = 175
CTL	*J_CTL, ECM_* = 350	*J_CTL, RN_* = 350	*J_CTL, CTL_* = 350	γ*_CTL, Tgt_* = 0
Tgt	*J_Tgt, ECM_* = 350	*J_Tgt, RN_* = 350	*J_Tgt, CTL_* = 350	*J_Tgt, Tgt_* = 350

*The terms ECM, CTL, Tgt, and RN, respectively, represent the extracellular matrix, cytotoxic T lymphocyte, target cell, and reticular network*.

### Mathematical Models

According to the double saturation (DS) model, the number of cells killed over a period Δ*t* is given by

(2) $$K_{\text{DS}}=k\prime \Delta t\frac{\bar{E}\bar{T}}{1+\bar{E}/h_{E}+\bar{T}/h_{T}},$$

where *k*′ is the mass-action killing rate; *h_E_* and *h_T_* are saturation constants in CTLs and target cells, respectively; and $\bar{E}$ and $\bar{T}$ are the number of CTLs and targets, respectively. For monogamous killing, both the saturation constants are the same, i.e., *h_E_* = *h_T_* = *h*. The resulting DS model is symmetric in CTL and target cell densities, which we refer to as the Padé model because it can be derived using a Padé approximation (8, 14). At low densities of CTLs and target cells, the total killing rate approaches the mass-action term, $k\prime \bar{E}\bar{T}$, showing that the killing rate *k*′ = *k*/*h* is a mass-action killing rate.

Whenever a fitting procedure leads to parameter estimates where *h_E_* → ∞ and *h_T_* → ∞ one should conclude that the data are well described by a mass-action process. If *h_E_ → ∞*, the DS model reduces to

(3) $$K_{\text{DS}|{\text{h}}_{\text{E}}\to \infty }=k\prime \Delta t\frac{\bar{E}\bar{T}}{1+\bar{T}∕h_{T}},$$

which is analogous to a conventional Holling’s type II functional response of predator–prey interactions in ecology (15), and if *h_T_ → ∞*, the DS model reduces to

(4) $$K_{\text{DS}|{\text{h}}_{\text{T}}\to \infty }=k\prime \Delta t\frac{\bar{E}\bar{T}}{1+\bar{E}∕h_{E}}.$$

### Statistical Analysis

The differences in the cumulative conjugate durations or the number of cells that neighbors CTLs and target cells observed in cube and slab configurations are examined using a chi-squared test. All the regression analyses of models to the simulated CPM data are performed using the function *nlinfit* in MATLAB (The MathWorks, USA), which uses the Levenberg–Marquardt algorithm. To prevent the fit to skew toward high number of cells killed, log-transformed numbers of cells killed were used for all the regressions.

## Results

### Cellular Potts Model Simulations of CTL-Mediated Killing

The CPM is a lattice-based model (9, 10), in which each cell is composed of multiple lattice sites. Previously, we performed 2D CPM simulations to determine the general functional response of CTL-mediated killing (8). Here, we investigate the quantitative differences of the CTL-mediated killing functional response between 2D and 3D environments.

**Figure 1:**
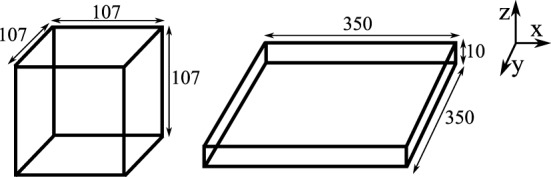
**Schematic of the fields**. The cube (left) and slab (right) are of equal volume, and the numbers on the edges indicate the dimensions (in micrometers) of the environments.

**Figure 2:**
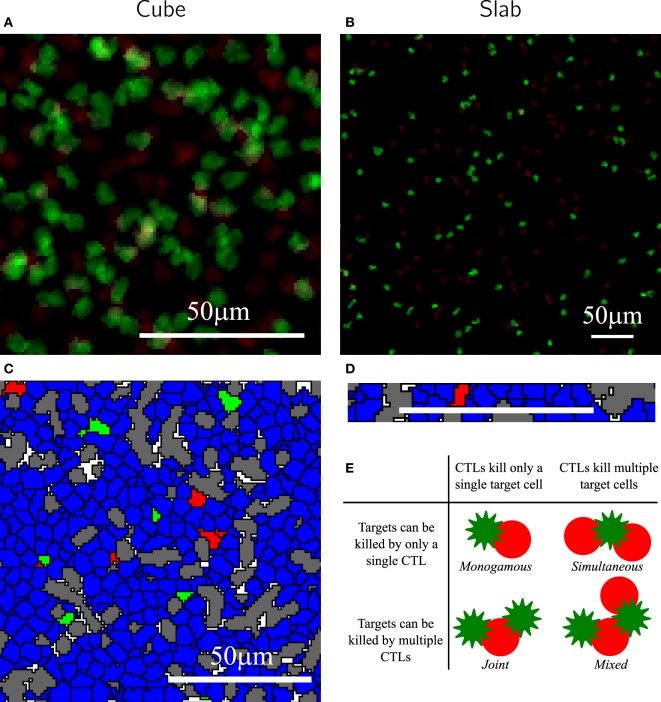
**Representative snapshots of the simulations in cube and slab**. Panels **(A,B)** are representative mean-intensity projections of the cube **(A)** and slab **(B)**, showing only the CTLs. Panels **(C,D)** are cross-sectional views of cube **(C)** and slab **(D)** simulations. To better visualize the cells of the slab, only a part of its cross-sectional snapshot is shown. **(E)** Illustration of the scenarios of killing. In all images, CTLs and target cells are shown in green and red, respectively, and the reticular network is shown in gray. Non-specific target cells and CTLs are shown in blue. These snapshots were taken from simulations with $\bar{E}=\text{100}$ CTLs and $\bar{T}=\text{100}$ targets. The scale bar indicates 50 *μ*m.

A direct comparison of the functional responses obtained from 2D and 3D simulations is difficult because of differences in the CPM simulation parameters, such as adhesion energies and “temperature” between 2D and 3D simulations. Specifically, the parameters in our previous 2D CPM simulations (8) were chosen such that the motility properties of *in silico* cells mimic those observed *in vivo* (2, 5). To achieve a similar motility in 3D simulations, we require a different set of CPM parameters, confounding a direct comparison of killing rates observed in 2D and 3D simulations. Instead, to rigorously compare the functional responses of CTL-mediated killing in different spatial environments, we consider two 3-dimensional fields of equal volume: a slab and a cube, with dimensions of 107 *μ*m × 107 *μ*m × 107 *μ*m and 350 *μ*m × 350 *μ*m × 10 *μ*m, respectively (Figure 1; a representative movie of the simulation in cube configuration is shown in Video S1 in Supplementary Material). The slab configuration resembles a 2D space as its height is close to the diameter of the cells in our simulations, and hence it mostly consists of cells in a monolayer. To restrict the migration of cells in the z-direction, we made the boundaries of both cube and slab fields in the z-direction impermeable to cells (i.e., a fixed boundary condition in the z-direction alone; see Figures 2A–D for representative snapshots of the simulations). Thus, the slab closely resembles a 2D-like space as for instance in skin epidermis, and the cube closely resembles a 3D space as in T cell areas of lymphoid tissues.

The simulation protocol used in this study is the same as in our previous study (see Methods). Briefly, we consider a finite 3D space (wrapped in the x- and y-directions), filled with static cylindrical rods representing the fibroblastic reticular network (RN), CTLs, and (migratory) target cells. The empty positions on the lattice represent extracellular matrix (Figures 2A–D). The configurations differ only in the dimensionality of the space; the rest of the parameters, including the volume of the field, total number of cells, and RN density, remain the same. CTLs and target cells perform a persistent random walk in this space according to well-defined migration rules [for details, see Methods and Ref. (8, 16, 17)]. The simulation parameters are chosen such that we approximate the migration properties of T cells observed in imaging studies (2). The migration speeds of CTLs and targets are similar between slab and cube: the average migration speeds of CTLs and targets are, respectively, 5.0 and 3.9 *μ*m/min in both the configurations (for migration speeds of representative cells, see Figure S1 in Supplementary Material). However, despite the same simulation parameters between slab and cube simulations, cells in the two configurations exhibit slightly different motility coefficients (Table 2). This could be because cells in a cube have the additional freedom to migrate in the z-direction, resulting in a lower motility coefficient in a cube simulation. Note that for the estimation of the motility coefficient by Fuerth’s equation (18), it is unclear whether a slab should be considered as a 2D or 3D space.

**Table 2:** **Summary of the migration properties of CTLs and targets observed in the two configurations**.

	Motility coefficient (*μ*m^2^/min)		Persistence time (min)	
	CTL	Target	CTL	Target
Cube	29.9 (29.84, 29.96)	17.4 (17.3, 17.5)	3.4 (3.3, 3.5)	3.3 (3.2, 3.4)
Slab (with *n* = 3)	34.4 (34.2, 34.6)	21.1 (21.0, 21.2)	3.9 (3.8, 4.0)	3.8 (3.75, 3.85)
Slab (with *n* = 2)	51.6 (51.3, 51.9)	31.7 (31.6, 31.8)	3.9 (3.8, 4.0)	3.8 (3.77, 3.83)

*Motility coefficients, *M*, and persistence times, *p*, are obtained by fitting Fuerth’s equation to the mean square displacement (*msd*) of cells: *msd* = 2*nM*{*t* − *p*(1 − *e*^−^^*t*/*p*^)}, where *t* is the time from the beginning of the observation and *n* is the dimensionality of the space (see Figure S1 in Supplementary Material). Numbers in parentheses indicate the 95% confidence intervals of the parameters*.

To quantitatively compare the functional response of CTL-mediated killing in slabs and cubes, we perform simulations with different numbers of antigen-expressing target cells, $\bar{T}$, and their cognate CTLs, $\bar{E}$, for four different killing scenarios: monogamous, joint, simultaneous, and mixed killing (Figure 2E). In the monogamous scenario, a CTL can only kill a single target cell at a time and a target cell can only be killed by a single CTL. The mixed killing scenario is opposite to monogamous, i.e., conjugates of multiple CTLs and multiple targets are allowed to form, in which each CTL can induce death of multiple target cells simultaneously and a target cell can be killed by multiple CTLs. Joint and simultaneous killing regimes are intermediates between monogamous and mixed regimes (Figure 2E). In joint killing, a CTL can kill a single target cell at a time, but a target cell can be killed by many CTLs acting together at the same time (19); whereas in the simultaneous regime, a CTL can induce death of multiple target cells simultaneously (20) and a target cell can only be killed by a single CTL. Unless otherwise specified, we do not restrict the number of conjugates that cells can form (for simplicity referred to as “binding sites” hereafter) in the non-monogamous regimes of killing and use a time required for a CTL to kill a target cell, *t_D_*, of 15 min (2). To our knowledge, there are no studies quantitatively comparing the times required by CTLs to induce target cell death during monogamous or non-monogamous killing. Therefore, in our simulations, we considered multiple CTLs bound to a target to independently induce its death (i.e., the time required to kill a target cell is inversely related to the number of CTLs in the conjugate). Similarly, we consider single CTLs bound to multiple targets to kill all of them within the same kill time, i.e., killing of individual targets remains the same irrespective of how many targets a CTL is conjugated with. Together, this represents the best-case scenario from the perspective of CTLs. To precisely determine the CTL killing rates at a particular CTL and target cell density, we maintain constant target cell numbers throughout the simulations, by immediately replacing every killed target cell with a new target cell at a random position in the field. CTLs in our simulations neither die nor divide.

**Figure 3:**
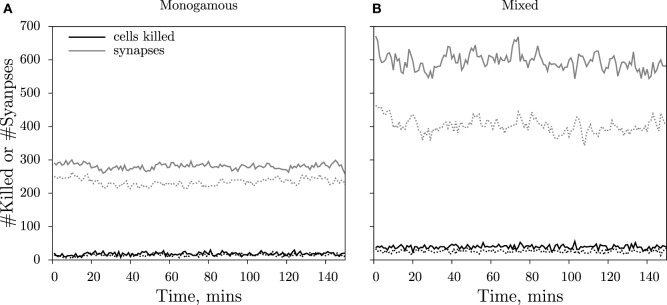
**Dynamics of cells killed and cytotoxic synapses**. The number of cells killed over 1-min intervals (black lines) and the total number of cytotoxic synapses (gray lines) during a simulation with $\bar{E}\;=\;\mathrm{500}$ CTLs and $\bar{T}\;=\;500$ targets for monogamous **(A)** and mixed killing **(B)**. The measurements from the cube simulations are depicted as solid lines, and those from the slab are depicted as dashed lines. For further processing, the numbers of cells killed over the last 75 min of the simulations are used to ensure that killing occurs at steady state.

In the simulations, we record the number of cells killed over intervals of 1 min and the total number of conjugates. Both measures approach a steady-state value soon after the start of the simulation (Figure 3). Because conjugated cells can form additional contacts with other cells during mixed killing, we observe more conjugates than in the monogamous scenario (compare Figures 3A,B). Furthermore, we consistently measured a higher number of conjugates in cube than in slab simulations (compare solid and dashed lines in Figure 3). Because killing takes 15 min of contact time, and because the number of cells killed is counted over intervals of 1 min at any given time, the number of cells killed is about 1/15 of the total number of conjugates (Figure 3). We perform simulations corresponding to 150 min in real time and count the number of cells killed during the last 75 min, i.e., when the system has approached a steady state. The 3D simulations are computationally expensive, with each simulation requiring about 8 days of CPU time on a single Intel Xeon processor, 3.33 GHz, with 48 GB of memory. For this reason, we limited our analysis to a single run for each CTL and target cell frequency.

### Conjugates Break Frequently in 3D Simulations

We allow conjugate formation in our simulations by incorporating a strong adhesion between CTL and target pairs upon their encounter and making the cells in a conjugate immotile (i.e., by setting *μ* = 0; referred to as the “stopping-rule” henceforth). Despite the enhanced adhesion and stopping rule, and contrary to our published 2D simulations (8), the conjugates in both cube and slab simulations are relatively unstable, i.e., they resemble the short-lived cytotoxic kinapses described in Ref. (21, 22). Apart from the videos, this can also be seen from the “killing signal” distribution: for monogamous simulations with targets in excess of CTLs (e.g., $\bar{E}=100$, $\bar{T}=1500$), the number of targets that are accumulating killing signal would be similar to the number of CTLs if the conjugates were stable. Instead, we observe that 10-fold more targets accumulate killing signal than there are CTLs present in the field (Figure S2 in Supplementary Material), implying that the conjugates dissociate frequently.

Targets “remember” the accumulated killing signal when a conjugate breaks, and upon renewed contact with another or with the same, CTL, the killing signal accrues on the existing signal. Since the targets remember that signal, these relatively unstable conjugates mimic a “multi-stage killing” scenario in which targets need to transit through multiple killing stages before being killed. Such multistage killing results in higher saturation constants in both CTL and target cell densities compared to “single-stage killing” in which killing is finished off by CTLs during a single interaction (see Appendix). Thus, we expect generally higher saturation constants in these 3D simulations relative to our earlier 2D simulations. Because the killing signal distributions, and therefore the durations of the conjugates, are highly similar between slab and cube configurations (*P* = 0.8, χ^2^ = 5.6; Figure S2 in Supplementary Material), we are assessing the influence of dimensionality in similar scenarios with short-lived kinapses. Note that we do not attempt to match the multistage killing scenario quantitatively to the data by Halle et al. (21).

### Monogamous Killing

**Figure 4:**
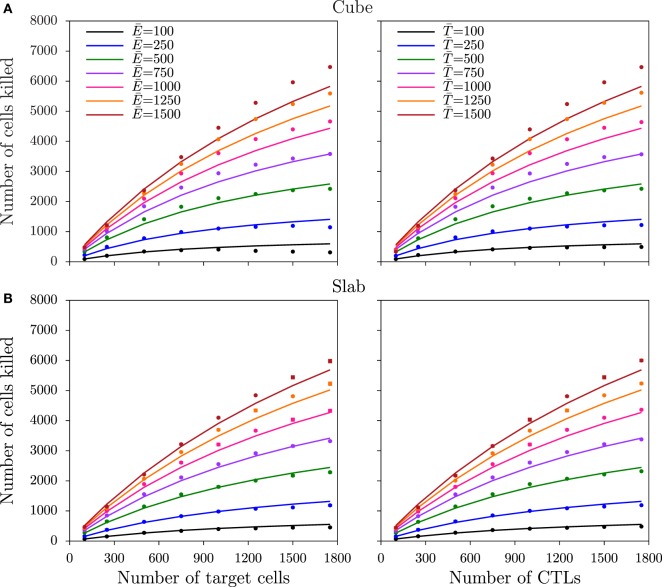
**Number of target cells killed for monogamous killing**. The total number of cells killed over the last 75 min of the simulations as a function of target cell (left panels) and CTL (right panels) densities, obtained from simulations in cube **(A)** and in slab **(B)** configurations. Markers depict the measurements from the simulations, and solid lines represent the DS model predictions with the best-fit parameters (Table 3).

In the monogamous killing regime, conjugates of just one CTL and one target cell are allowed to form (Figure 2E). As expected (8, 23), also in the 3D slab and cube configurations, the number of cells killed saturates symmetrically with an increase in CTL and target cell frequencies (Figure 4). The saturation in killing is expected from the handling time (which in our case is *t_D_* = 15 min) (23, 24), and its symmetric nature is due to the monogamous CTL–target interactions (8, 23, 24). The functional response for monogamous killing can be derived by applying an enzyme–substrate analogy in which either only a Quasi-Steady-State Approximation (QSSA) is employed or first a QSSA and subsequently a Padé approximation, which simplifies a complex to an approximate rational function (14, 23), resulting in *K*_QSSA_ or *K*_Padé_, respectively:

(5) $$\begin{array}{ll} K_{\text{QSSA}} & =k_{2}\Delta t\frac{h+\bar{E}+\bar{T}-\sqrt{{\left(h+\bar{E}+\bar{T}\right)}^{2}-4\bar{E}\bar{T}}}{2},\\ K_{\text{Pad}é} & =k\prime \Delta t\frac{\bar{E}\bar{T}}{1+\bar{E}∕h+\bar{T}∕h}, \end{array}$$

where $\bar{E}$ and $\bar{T}$ are the total number of cognate CTLs and target cells, respectively; *k*_2_ is the killing rate of conjugates; *h* is the Michaelis–Menten constant, defined as $\frac{k_{2}+k_{-1}}{k_{1}}$; *k*_1_ and *k*_−__1_ are the rates at which conjugates form and dissociate, respectively; and *k*′ in the DS model is the mass-action killing rate (8), defined as *k*_2_/*h*. Note that these equations can also be derived for multistage killing (see Appendix). Fitting the DS and full QSSA models to the simulated data from the two spatial configurations, we find that the full QSSA model describes the data better than the Padé model (compare Figure S3 in Supplementary Material to Figure 4), particularly when the numbers of CTLs and targets together are larger than 40% of the total cell number in the field. For biological scenarios, this minor discrepancy is not very important because CTL frequencies remain limited. For example, to deal with malaria infections, a threshold CTL frequency of about 20% has been shown to be sufficient for protection (25), which is well below the CTL frequencies we simulated. Nevertheless, the QSSA model should be used if the Padé model fails to describe the killing at high cell densities and if the CTL–target cell interactions are monogamous. These results are consistent with our published 2D simulations (8). Interestingly, the saturation in killing sets in at higher CTL and target cell densities in the slab configuration, compared to the cube (i.e., the saturation constant is about 1.6-fold lower in cube; see Table 3). Thus, the spatial configuration affects the functional response of CTL-mediated killing.

**Table 3:** **Best-fit parameters and 95% confidence ranges of the double saturation (DS) model for the different killing regimes in slab (A) and in cube (B) configurations**.

(A) Slab	*k*′(cells^−1^ min^−1^)	*h_E_* (cells)	*h_T_* (cells)
Monogamous	1.07 (±0.07) × 10^−4^	*h_E_* = *h_T_* = 1,202 (±137)	
Joint	1.18 (±0.07) × 10^−4^	∞	740 (±88)
Simultaneous	1.18 (±0.04) × 10^−4^	889 (±73)	∞
Mixed	1.08 (±0.01) × 10^−4^	*h_E_* = *h_T_* → ∞	
**(B) Cube**			
Monogamous	1.54 (±0.29) × 10^−4^	*h_E_* = *h_T_* = 772 (±216)	
Joint	1.71 (±0.12) × 10^−4^	∞	446 (±49)
Simultaneous	1.86 (±0.08) × 10^−4^	482(±35)	∞
Mixed	1.61 (±0.02) × 10^−4^	*h_E_* = *h_T_* = 3.8 (±0.95) × 10^4^	

*For cases where there is no evidence for saturation, the saturation constant is marked with ∞*.

The slab and cube configurations are both 3D fields composed of the same total number of CTLs and target cells and of the same total volume. Therefore, the saturation constants can be directly compared between the two spatial configurations. The differences in the saturation constants between slab and cube could either result from altered migration properties or from altered CTL–target encounter rates, both emerging due to differences in the spatial organization. As the migration properties are only slightly different between the two configurations (Table 2), we hypothesized that the higher killing in cube simulations is due to a faster detection of target cells, i.e., a higher rate of conjugate formation, *k*_1_. Since the diameter of the CTLs and targets is comparable to the height of the slab, CTLs in the slab are expected to scan fewer cells compared to CTLs in the cube configuration. Indeed, at each time point, the number of targets that are in contact with each CTL in simulations with an excess of target cells is higher in a cube than in a slab configuration (e.g., $\bar{E}=100,\;\bar{T}=1500$ cells: Figure S4 in Supplementary Material; *P* < 0.01, χ^2^ = 95). Similarly, the number of neighboring CTLs per target is highest in cube simulations (not shown). Taken together, the CTL–target encounter rates are highest in cube simulations, which will result in an increased rate of conjugate formation, *k*_1_, and in a higher number of conjugates in cube compared to slab simulations (Figure 3). As a result, fewer CTLs are sufficient to achieve the maximal killing rate in a cube, i.e., killing saturates at lower cell densities than in a slab configuration (*h* = (*k*_2_ + *k*_−__1_)/*k*_1_).

### Simultaneous and Joint Killing

**Figure 5:**
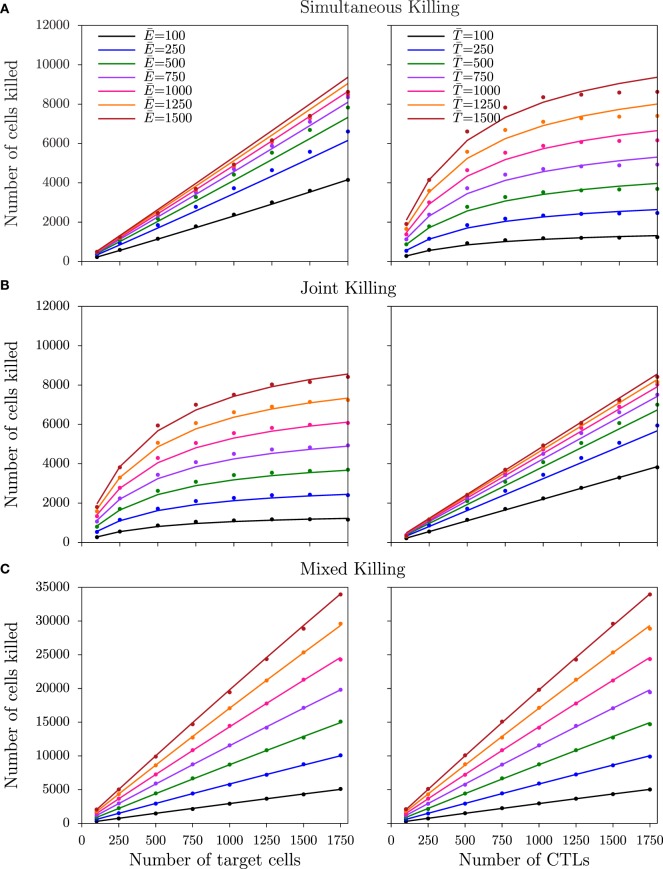
**Number of target cells killed for non-monogamous killing regimes in the cube configuration**. The number of cells killed as a function of CTL and target cell densities for simultaneous **(A)**, joint **(B)**, and mixed **(C)** killing regimes in cube simulations. Markers indicate the total number of cells killed over the last 75 min of simulations, and solid lines represent the predictions of the DS model with the best-fit parameters (Table 3). Functional responses observed in the slab simulations are shown in Figure S5 in Supplementary Material.

In the simultaneous killing regime, a CTL can kill multiple target cells simultaneously, but a target cell can only be killed by a single CTL (Figure 2E). As for monogamous killing, the functional response can be analytically derived following a QSS assumption (8) and is given by

(6) $$K_{\text{QSSA}}=\frac{k_{2}\Delta t}{2}\left(m\bar{E}+h+\bar{T}-\sqrt{{\left(m\bar{E}+h+\bar{T}\right)}^{2}-4m\bar{E}\bar{T}}\right),$$

where *k*_2_ is the killing rate, *m* is the maximum number of targets bound to a CTL (i.e., binding sites on a CTL), Δ*t* is the time period during which killing is measured, and *h* is the Michaelis–Menten-like saturation constant, defined as (*k*_2_ + *k_−_*_1_)/*k*_1_ (8). This can again be simplified using a Padé approximation to a double saturation (DS) model with two different saturation constants—one for CTL and another for target cell densities:

(7) $$K_{\text{DS}}=\frac{mk_{2}\bar{E}\bar{T}\Delta t}{h+m\bar{E}+\bar{T}}=\frac{k\prime \bar{E}\bar{T}\Delta t}{1+\bar{E}∕h_{E}+\bar{T}∕h_{T}},$$

where *k*′ is the mass-action killing rate, defined as *mk*_2_/*h*, and *h_E_* = *h*/*m* and *h_T_* = *h* are the saturation constants in CTLs and target cells, respectively. From this equation derived for simultaneous killing, the number of cells killed is expected to saturate at lower CTL densities than target cell densities (i.e., asymmetric saturation), which is confirmed in both cube (Figure 5A) and slab configurations (Figure S5A in Supplementary Material). For joint killing, we have been unable to derive the functional response analytically. Nevertheless, the DS model with two different saturation constants in CTLs and targets provides a semi-mechanistic description of joint killing (8). Indeed, in both slab and cube simulations, the number of cells killed saturates in target cell frequencies but increases linearly with CTL frequencies (i.e., the converse of simultaneous killing; Figure 5B; Figure S5B in Supplementary Material).

As expected from visual inspection of the DS model fits of equation (7) and of equation (2) (see Methods) to, respectively, the data from simultaneous and joint killing in cube and slab simulations, in all cases one of the saturation constants approaches infinity (i.e., an estimate much larger than the CTL and target cell numbers used). Indeed, the DS model does in these cases not improve the description of the data over a model with a single saturation constant [simultaneous killing: *P* = 0.08, *F*_1,61_ = 3.09 (cube); *P* = 0.08, *F*_1,61_ = 3.09 (slab); joint killing: *P* = 1, *F*_1,61_ ≈ 0 (cube and slab); Table 3], suggesting a lack of saturation within this range of cell densities examined.

With respect to the comparison between the two spatial configurations, joint killing resembles monogamous killing in the onset of saturation at lower CTL densities in cube than in slab simulations, likely again due to fast target detection in cubes [i.e., increasing *k*_1_ in equations (6) and (7)]. Consistent with this, we find that the average number of targets in conjugate with a CTL is higher in cube than in slab simulations (not shown; *P* = 10^−12^ for a simulation with $\bar{E}=100$, $\bar{T}=1500$ cells).

### Mixed Killing

Next, we performed simulations for the mixed killing scenario in which CTLs can induce killing of multiple target cells simultaneously and target cells can be killed jointly by multiple CTLs (Figure 2E). Both in the slab and cube configurations, the number of cells killed increases almost linearly with target cell and CTL densities (Figure 5C; Figure S5C in Supplementary Material). Fitting DS and mass-action (*h_E_* = *h_T_* → ∞; see Methods) models to the data from mixed killing, we indeed find no evidence for saturation in the slab configuration (*P* = 1, *F*_1,62_ = 0), but a late—though significant—saturation in cube configurations (*P* < 10^−11^, *F*_1,62_ = 71). Thus, the late saturation in our previous 2D simulations of the mixed killing scenario (8) is further reduced in the cube configuration and is completely absent in the slab configuration. Since conjugates were breaking at a similar frequency in the cube and slab simulations, which is evident by the killing signal distribution of the targets (not shown), the differences in the saturation between the two configurations are due to the higher CTL–target encounter rates in cube simulations.

Two factors may explain why we find less saturation in the current 3D mixed killing simulations compared to our previously published 2D simulations. First, conjugates are less stable now, and the associated breaking of conjugates is expected to delay the onset of saturation (see Appendix). Second, because of the higher surface area of cells in 3D simulations compared to 2D (8), the maximum number of possible binding sites on cells in 3D is higher than that in 2D, which also delays the onset of saturation (8). Indeed, when we restricted the maximum number of cells in a conjugate, saturation is more pronounced compared to unrestricted binding (Figure S6 in Supplementary Material). In summary, the later saturation in 3D compared to 2D is due to a combination of a high number of binding sites and short-lived conjugates.

A comparison of all the four killing regimes within a spatial configuration shows that the best-fit *k*′ values differ between cube and slab configurations but are similar across the killing regimes. Because *k*′ is equivalent to the mass-action killing rate (see Methods), this shows that the CTL–target encounter rates are similar across all the killing regimes. Moreover, such equality of encounter rates is a prerequisite for a fair comparison across regimes with respect to the cell densities at which the killing efficiency saturates. Taken together, our results show that the DS model is qualitatively robust to variation in spatial factors and that dimensionality of the space plays a role in determining at which cell densities saturation starts.

## Discussion

By simulating CTL killing in two distinct 3D configurations, we have shown that the total number of target cells killed per day saturates when either the density of target cells or that of CTLs becomes large. Consistent with our previously published 2D simulations (8), in joint and simultaneous regimes, i.e., the asymmetric killing scenarios, the saturation is most pronounced for either CTLs or targets, respectively. Similarly, killing saturates symmetrically with CTL and target cell frequencies if their interactions are symmetric (i.e., monogamous and mixed regimes). We conclude that the double saturation (DS) function remains a robust functional response describing CTL-mediated killing, also in different spatial configurations.

The CPM is one of the most sophisticated spatial modeling formalisms available to model cellular interactions, and it has been used to answer a variety of questions on topics, such as cell migration (16), morphogenesis (26), and tissue development (27). However, one of the limitations in these 3D CPM simulations is that conjugate stability can be difficult to achieve. For a given diameter of a cell, the number of neighbors that a cell can have in 3D spaces is greater than in 2D spaces. As a result of the pressure of many migratory neighboring cells, the cells in a conjugate can be pushed apart, resulting in a reduced stability of the conjugates in 3D compared to 2D. A consequence of such frequent conjugate dissociation is that the onset of saturation in killing (when combined with target cell memory of previous killing signal) is delayed. Therefore, the high saturation constants in the 3D simulations are partly due to the relatively unstable conjugates.

Because the killing signals that the targets accumulated were similar between slab and cube simulations—showing that the conjugates break at similar frequency in cube and slab simulations—the comparison of the functional response between the two configurations remains unaffected by the relatively short-lived conjugates. Further, as we only vary the spatial dimensions of the fields between the two configurations, the differences in the functional response of killing arise from the different search efficiencies that emerge between the two configurations, thereby highlighting the importance of tissue dimensionality. Earlier saturation in cube compared to slab simulations implies that the maximal killing rate is attained with fewer CTLs, suggesting that CTLs kill most efficiently in 3D environments within which they can migrate in an unrestricted manner. To resolve infections and tumors in 3D, boosting the CTL numbers by using a vaccine or by adoptive T cell transfer may not help when the killing rate is already close to the maximal level. Hence, this is not because CTLs are inefficient in 3D, but because the maximal killing rate is already achieved and cannot further be enhanced by the presence of even more CTLs. In such cases, therapies should aim to increase the quality of the CTL–target interactions that lead to killing (e.g., by replacing T cell receptors with chimeric antigen receptors) rather than increasing the number of CTLs further.

The short-lived conjugates in our 3D simulations are actually quite realistic because recent *in vivo* experiments using intravital two-photon microscopy revealed that virus-infected cells often break their contact with CTLs and tend to be killed during subsequent conjugates with other CTLs (21, 22). In these experiments, CTLs rarely formed stable conjugates and remained motile after contacting a target cell. The probability of death of infected cells increased for targets contacted by more than two CTLs, which was interpreted as evidence for CTL cooperation (21). Similarly, within *in vitro* collagen gel experiments about 50% of the HIV-infected CD4^+^ T cells remained motile and broke their conjugate with CD8^+^ T cells (28).

Our results predict variation in the killing efficiency within different environments *in vivo*. For instance, CTLs specific to human immunodeficiency virus colocalize with infected cells in the subcapsular sinus of LNs (1). Since sinuses of LNs are narrow spaces, they roughly resemble our slab configuration. Even when all other conditions are similar, our findings suggest that the CTL-mediated killing of HIV-infected cells in this narrow spatial environment is less efficient than the killing of peptide-pulsed B cells, which predominantly occurs in 3D-like T cell areas (2).

In summary, our results suggest that the spatial configuration of the environment may play a role in determining the extent of saturation in CTL-mediated killing. Moreover, we find that the DS model describes the data in different spatial configurations very well, unless the CTL and target cell frequencies are extremely high. Thus, the double saturation model is a reliable general functional response of CTL-mediated killing, that is, able to capture different spatial environments.

## Author Contributions

SG, JB, and RB conceived and designed the study, developed the methodology, performed the analysis, and wrote the manuscript. SG performed the simulations. AM developed the simulation environment.

## Conflict of Interest Statement

The authors declare that the research was conducted in the absence of any commercial or financial relationships that could be construed as a potential conflict of interest.

## Supplementary Material

Video S1 **Representative movie of a cube simulation with $\bar{E}=\text{500}$ and $\bar{T}=\text{500}$**. CTLs and target cells are shown in green and red, respectively, and the reticular network is shown in gray. Non-specific target cells and non-specific CTLs are shown in blue.

## Appendix

### Functional Response of Multistage CTL-Mediated Killing

To study the effect of multistage killing, as in our CPM simulations, the total time a target spends in a conjugate before being killed (i.e., *t_D_*) should be the same for single- and multistage killing (i.e., irrespective of the number of stages). For *n* stages, this is achieved by setting $k\prime_{2}=nk_{\text{2}}$. In this modified model, targets transit *n* sequential stages of conjugate before being killed:

(A1) $$\begin{array}{c} E+T\underset{k_{-1}}{\overset{k_{1}}{⇌}}\;ET_{0}\overset{nk_{2}}{\to }\;E+T_{1},\\ E+T_{1}\underset{k_{-1}}{\overset{k_{1}}{⇌}}\;ET_{1}\overset{nk_{2}}{\to }\;E+T_{2},\\ \vdots \\ E+T_{n-1}\underset{k_{-1}}{\overset{k_{1}}{⇌}}\;ET_{n-1}\overset{nk_{2}}{\to }\;E+T^{*}, \end{array}$$

where *E*, *T*, *T_j_*, and *T** represent free cognate CTLs, naïve target cells, partially lysed targets, and dead targets, respectively; [*ET_j_*] (*j* = 0 to *n* − 1) represent the conjugates in the stage *j* + 1 of killing, respectively; *k*_1_ and *k*_−__1_ are the rates of conjugate formation and dissociation, respectively; *nk*_2_ is the rate at which targets transit each stage; and *k*_2_ is the killing rate during singlestage killing.

Similar to earlier studies (6, 8), we make a quasi-steady-state approximation (QSSA) for all [*ET_j_*] (*j* = 0 to *n* − 1) to derive an expression for the killing rate [see Gadhamsetty et al.^1^ for a full derivation]. We obtain the number of target cells killed over a time period Δ*t*, computed by full and Padé approximation, respectively, as

(A2) $$K_{\text{full}}\;=\;nk_{2}C\Delta t\;=\;\Delta t\frac{k_{2}}{2}\left(h\prime \;+\;\bar{E}\;+\;\bar{T}\;-\;\sqrt{{\left(h\prime +\bar{E}+\bar{T}\right)}^{2}-\;4\bar{E}\bar{T}}\right),$$

(A3) $$K_{\text{Padé}}=nk_{2}C\Delta t=\Delta t\frac{k\prime \bar{E}\bar{T}}{1+\bar{E}∕h\prime +\bar{T}∕h\prime }.$$

From the definition of the saturation constant $h\prime =\left(nk_{2}+k_{-1}\right)∕k_{1}$, the saturation during multistage killing sets in at higher CTL and target cell densities than during singlestage killing, despite the same overall killing rate *k*_2_ as in singlestage killing. Because the saturation constant contributes to the mass-action killing rate (8), i.e., $k\prime =k_{2}∕h\prime$, the mass-action killing rate *k*′ also decreases for multistage killing.

^1^ Gadhamsetty S, Marée AF, Beltman JB, de Boer RJ. A sigmoid functional response suggesting ‘co-operation’ emerges when cytotoxic t lymphocyte start killing fresh target cells. *Biophys J* (Forthcoming).
