# Supplementary material for: Tissue Dimensionality Influences the Functional Response of Cytotoxic T Lymphocyte-Mediated Killing of Targets
Source: Front Immunol. 2017 Jan 11;7:668. doi: 10.3389/fimmu.2016.00668 (PMC5225319; doi:10.3389/fimmu.2016.00668)
Supplement: Supplementary file 1 [file image_1.pdf]

Supplementary figures

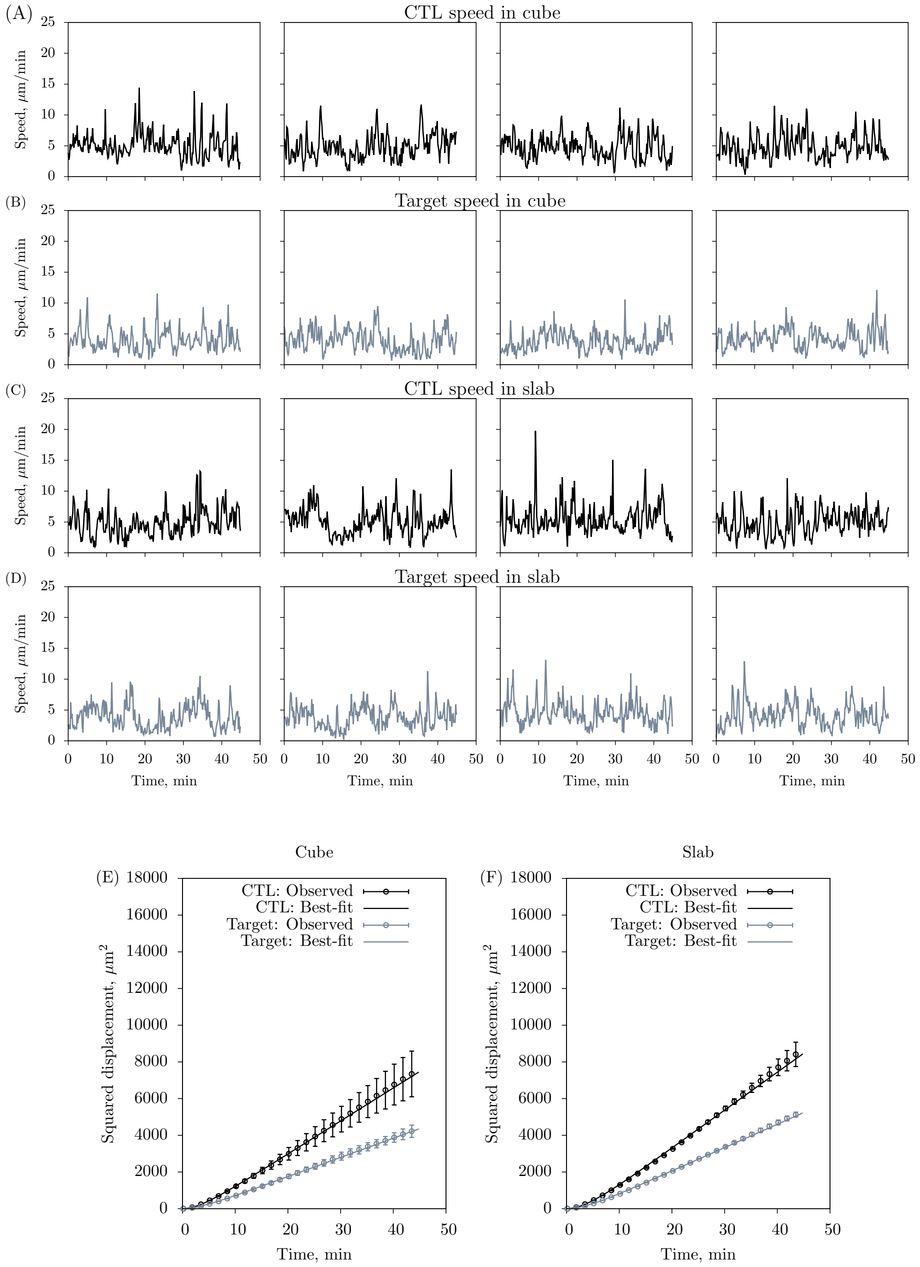

Figure S. 1: **Migration properties of individual CTLs and targets in the simulations.** Panels (A-D) show representative plots of migration speeds over time in cube (A-B) and slab (C-D) simulations (in the absence of killing): (A and C) four representative simulated CTLs, (B and D) four representative simulated target cells. Mean square displacement (msd) plots (in the absence of killing) for 3-dimensional simulations of (E) cube and (F) slab. Markers indicate the mean over four independent simulations, and solid lines depict the best-fit predictions of Fuerth's equation (18): $msd = 2nM\{(t - p(1 - e^{-t/p})\}$, where t is the time from the beginning of the observation, M is the motility coefficient, p is the persistence time and $n = 3$ is the dimensionality of the field. The migration properties of CTLs are depicted in black lines, and of targets in gray lines.
