# Supplementary material for: Tissue Dimensionality Influences the Functional Response of Cytotoxic T Lymphocyte-Mediated Killing of Targets
Source: Front Immunol. 2017 Jan 11;7:668. doi: 10.3389/fimmu.2016.00668 (PMC5225319; doi:10.3389/fimmu.2016.00668)
Supplement: Supplementary file 2 [file image_2.pdf]

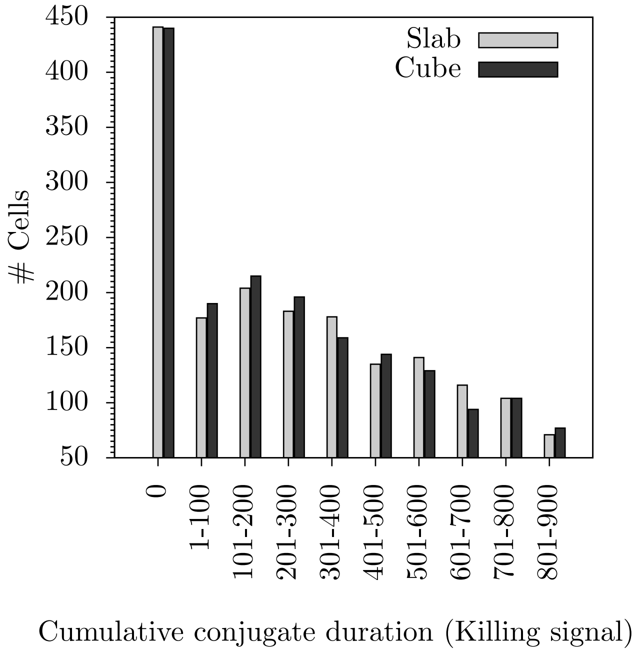

Figure S. 2: **Distributions of cumulative conjugate durations (killing signal) for all targets in monogamous simulations** ($\bar{E} = 100$, $\bar{T} = 1500$ cells). The killing signal distributions in the slab (gray bars) and in the cube (black bars) are categorized into bins, and are not significantly different ($P = 0.7$, $\chi^2 = 5.6$). Target cells are killed when their accumulated killing signal reaches 900 s (i.e., $t_D = 15$ mins).
