# Supplementary material for: Tissue Dimensionality Influences the Functional Response of Cytotoxic T Lymphocyte-Mediated Killing of Targets
Source: Front Immunol. 2017 Jan 11;7:668. doi: 10.3389/fimmu.2016.00668 (PMC5225319; doi:10.3389/fimmu.2016.00668)
Supplement: Supplementary file 3 [file image_3.pdf]

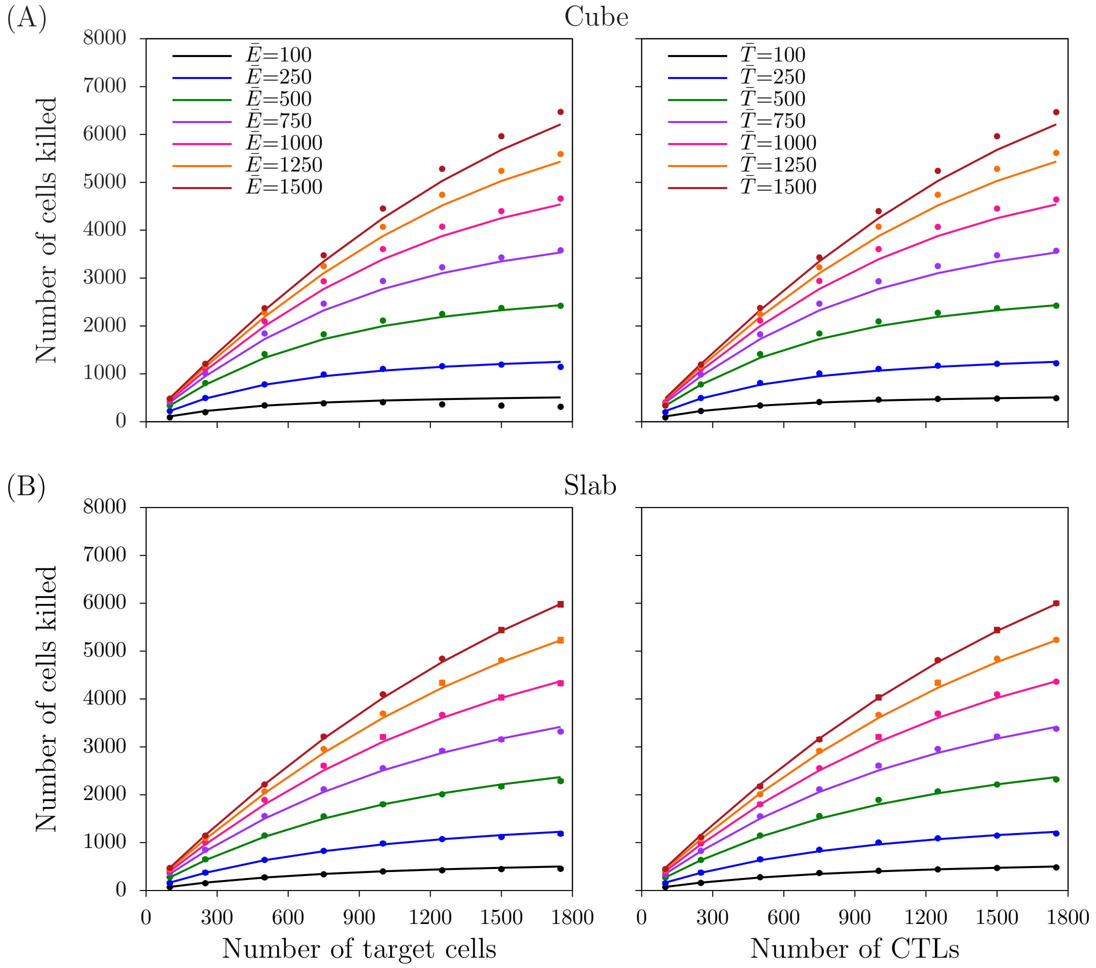

Figure S. 3: **Number of target cells killed during monogamous killing and fitted with the full QSSA model.** The total number of cells killed over 75 min of simulation as a function of target cell (left panels) and CTL (right panels) densities, obtained from simulations in a cube (A) and in a slab (B). Markers depict the measurements from the simulations, and solid lines represent the full QSSA model predictions with the best-fit parameters, slab: $k_2 = 9.8 \times 10^{-2}$ min^{-1}, $h = 792$ cells; cube: $k_2 = 8.3 \times 10^{-2}$ min^{-1}, $h = 381$ cells.
