# Supplementary material for: Tissue Dimensionality Influences the Functional Response of Cytotoxic T Lymphocyte-Mediated Killing of Targets
Source: Front Immunol. 2017 Jan 11;7:668. doi: 10.3389/fimmu.2016.00668 (PMC5225319; doi:10.3389/fimmu.2016.00668)
Supplement: Supplementary file 4 [file image_4.pdf]

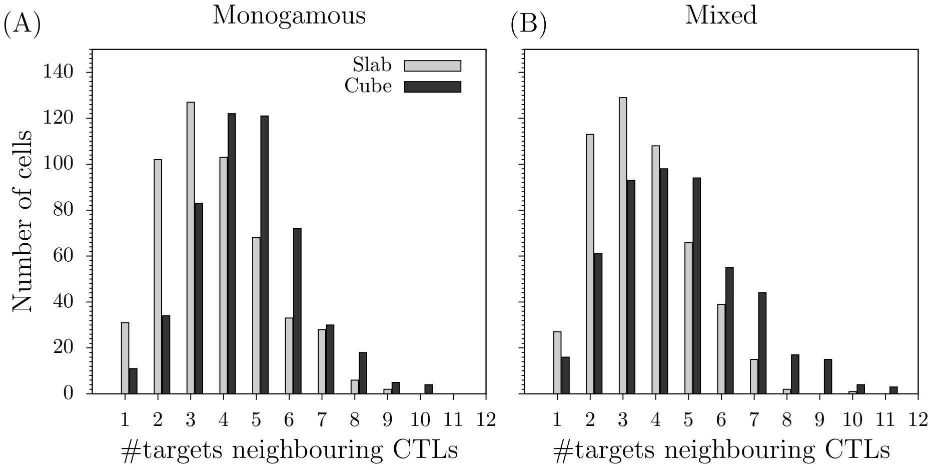

Figure S.4: **Distributions of CTLs with different numbers of contacting targets.** The number of neighboring targets observed during a monogamous killing simulation with $\bar{E} = 100$ and $\bar{T} = 2450$ in monogamous (A) and mixed (B) killing. Black bars indicate the number of neighboring targets observed in cube simulations, and gray bars in slab simulations. The difference between slab and cube simulations are significant ($P < 0.01$, $\chi^2 = 95$).
