# Supplementary material for: Tissue Dimensionality Influences the Functional Response of Cytotoxic T Lymphocyte-Mediated Killing of Targets
Source: Front Immunol. 2017 Jan 11;7:668. doi: 10.3389/fimmu.2016.00668 (PMC5225319; doi:10.3389/fimmu.2016.00668)
Supplement: Supplementary file 5 [file image_5.pdf]

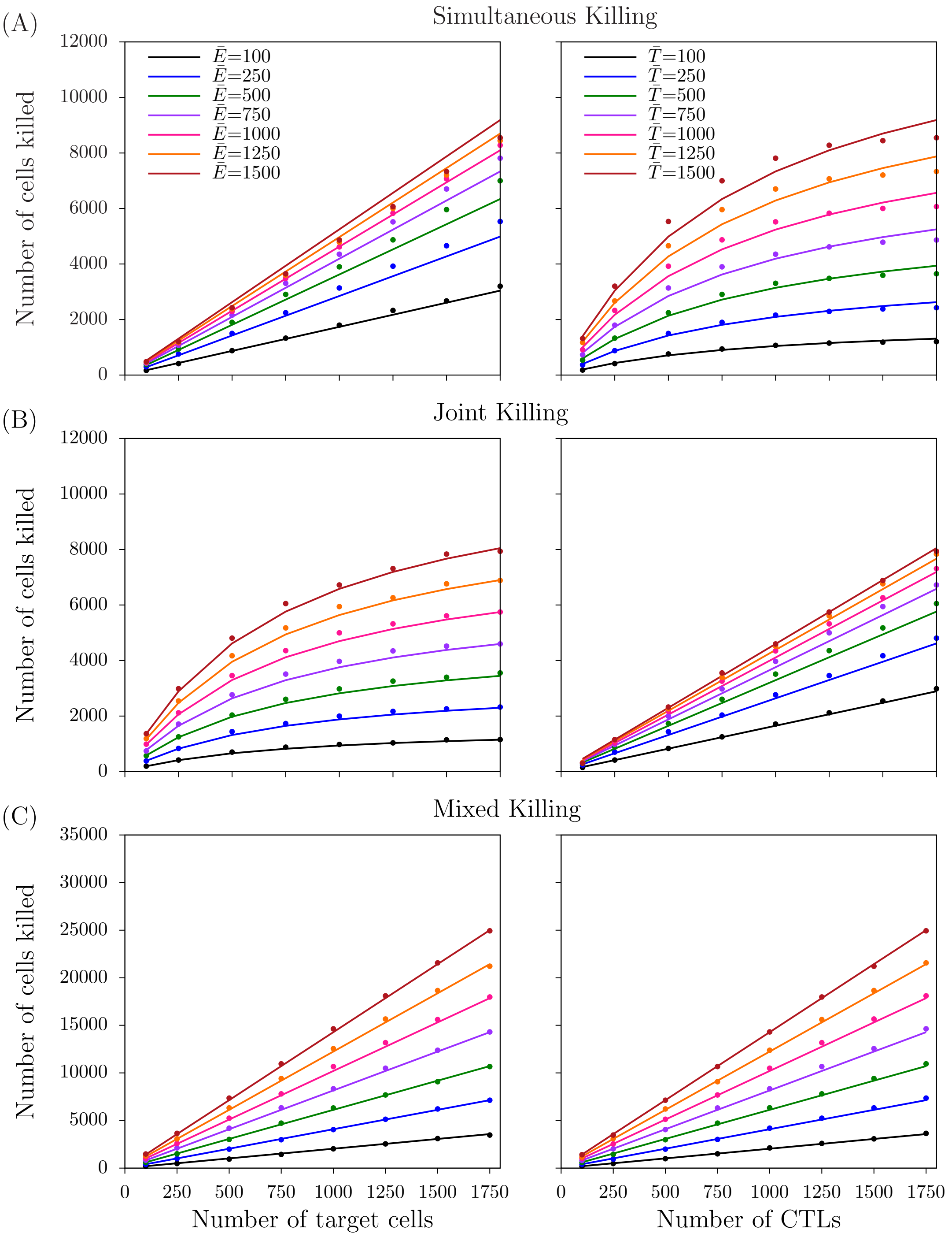

Figure S. 5: **Number of target cells killed for non-monogamous killing regimes in slab simulations.** The number of cells killed as a function of CTL and target cell densities for simultaneous (A), joint (B), and mixed (C) killing regimes. Markers indicate the mean of the total number of cells killed over 75 mins, and solid lines represent the prediction of the DS model with the best-fit parameters (Table 3).
