# Supplementary material for: Tissue Dimensionality Influences the Functional Response of Cytotoxic T Lymphocyte-Mediated Killing of Targets
Source: Front Immunol. 2017 Jan 11;7:668. doi: 10.3389/fimmu.2016.00668 (PMC5225319; doi:10.3389/fimmu.2016.00668)
Supplement: Supplementary file 6 [file image_6.pdf]

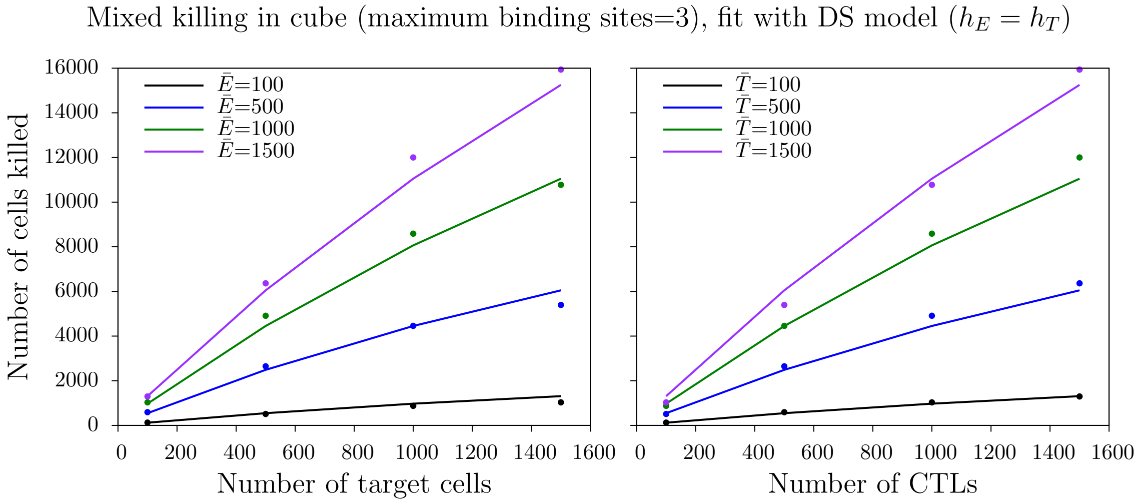

Figure S. 6: **Number of cells killed for mixed killing (in cube) with the maximum number of binding sites restricted to three.** Markers depict the measurements from the simulations, and solid lines represent the DS model predictions with the best-fit parameters: $k' = 1.73 \times 10^{-4}$ cells^{-1} min^{-1}, $h_E = h_T = 3.27 \times 10^3$ cells.
